# Context, attention, and the switch between habit and goal-direction in behavior

**DOI:** 10.3758/s13420-021-00488-z

**Published:** 2021-10-28

**Authors:** Mark E. Bouton

**Affiliations:** Department of Psychological Science, University of Vermont, 2 Colchester Ave, Burlington, VT 05405-0134 USA

**Keywords:** Habit, Goal-directed action, Context, Attention, Instrumental learning, Addiction

## Abstract

This article reviews recent findings from the author’s laboratory that may provide new insights into how habits are made and broken. Habits are extensively practiced behaviors that are automatically evoked by antecedent cues and performed without their goal (or reinforcer) “in mind.” Goal-directed actions, in contrast, are instrumental behaviors that are performed because their goal is remembered and valued. New results suggest that actions may transition to habit after extended practice when conditions encourage reduced attention to the behavior. Consistent with theories of attention and learning, a behavior may command less attention (and become habitual) as its reinforcer becomes well-predicted by cues in the environment; habit learning is prevented if presentation of the reinforcer is uncertain. Other results suggest that habits are not permanent, and that goal-direction can be restored by several environmental manipulations, including exposure to unexpected reinforcers or context change. Habits are more context-dependent than goal-directed actions are. Habit learning causes retroactive interference in a way that is reminiscent of extinction: It inhibits, but does not erase, goal-direction in a context-dependent way. The findings have implications for the understanding of habitual and goal-directed control of behavior as well as disordered behaviors like addictions.

## Introduction

The study of voluntary or instrumental behavior in the animal laboratory looks different now than it did some years ago. Although researchers still study rats and mice pressing levers for food rewards in Skinner boxes, it is now customary to imagine that the rodent is more cognitively engaged than classic behaviorists like Hull and Skinner assumed. In this article, I discuss a central distinction between two types of voluntary behavior that learning theorists now make, and explore a new perspective on how they might operate and interrelate. The distinction is that voluntary actions (instrumental or operant behaviors) come in two varieties: goal-directed actions and habits (Balleine, 2019; Dickinson, 1985; Everitt & Robbins, 2005, 2016; Robbins et al., 2019). Actions are instrumental behaviors that are emitted because they get us closer to a reward or a goal. As I illustrate below, they are apparently performed – and this is the “cognitively engaged” part – with a representation of the goal in memory. Habits, in contrast, are behaviors that may eventually get us to a goal, but are performed more automatically, without the goal in memory or “mind.” Actions can become habits with extended repetition and practice. The distinction has many connections with what social scientists and even popular writers have written about voluntary behavior in humans (e.g., Duhigg, 2012; Wood, 2019).

There is now clear evidence that a lever-pressing rat is not pressing because the food pellet has merely strengthened the response. If we separately “devalue” the reinforcer, for example by pairing it with sickness induced by an injection of a toxin (like LiCl), and thus condition a taste aversion to it, it changes the rat’s rate of making the response. Specifically, if we now test lever pressing in extinction, so that the pellet is never paired directly with the response after it has been averted, we find that the rat suppresses its lever pressing (e.g., Adams, 1982; Colwill & Rescorla, 1985) (See Table 1 for a summary of the design). This result – the *reinforcer devaluation effect*– suggests that the animal has learned that the behavior produces the food pellet, and that it makes the response only if it currently values the pellet. In the language of the field, the rat has learned to associate the response (R) with its outcome (O) (R-O), and performs R when it desires or values O. Reinforcer devaluation is also the main method used to identify habits; these are instrumental responses that are not affected by the devaluation of O. For example, if the lever-pressing rat is pressing out of habit rather than goal-direction, separate conditioning of a taste aversion to O has no influence on the response during the extinction test (e.g., Adams & Dickinson, 1981; Thrailkill & Bouton, 2015). The animal continues to press the lever, even though it would reject the pellet O if it were offered. The idea is that the animal has associated R with the prevailing stimuli or situation (S) in a classic S-R association. Informally, behavior is habitual if, in the presence of S, the animal makes the response without cognitively processing O.

**Table 1:** Reinforcer devaluation by taste aversion learning

Group	Operant training	Reinforcer devaluation	Extinction Test
Paired		O-LiCl	
	R-O		R?
Unpaired		LiCl, O	

*Note. R* Response, *O* Outcome (reinforcer), *LiCl* Lithium Chloride. In the Extinction Test, R is suppressed in the Paired group if the R is a goal-directed action. R is not suppressed if it is a habit. A second way of devaluing the reinforcer (not shown) is to satiate the animal on the O (compared to an irrelevant O2) just before the Extinction Test. If R is a goal-directed action (and not a habit) R is suppressed during the Extinction Test in the Paired group

The ability to form habits makes functional sense, because their automaticity presumably leaves cognitive capacity (e.g., working memory space) to process other things so that we can get around the world without fumbling over the routine. Of course, habits also have a dark side; despite their usefulness, they are considered by some to be important in the development of maladaptive behaviors like drug dependence, compulsions, and obsessive-compulsive disorders (e.g., Everitt & Robbins, 2005, 2016; Robbins et al., 2019; White, 1996; but see Hogarth, 2020; Vandaele & Ahmed, 2020). Consistent with a role for them in addictions, exposure to drugs of abuse can hasten the development of habit (or the loss of goal-direction) as measured by reinforcer devaluation techniques like the ones described above (e.g., Corbit et al., 2012; Corbit et al., 2014; Furlong et al., 2014; Furlong et al., 2018; Nelson & Killcross, 2006, 2013). The possibility that habits can contribute to addiction disorders is a powerful idea, and it potentially explains many features of addiction, including the sense that they seem out of voluntary control. However, although it is rarely argued in exactly this way, the habit perspective can be taken to imply that habit is a kind of behavioral *endpoint*. That is, the progression from action to habit with repetition and/or drug exposure seems permanent and fixed. Indeed, a recent quantitative model of habit acquisition assumes that habit learning replaces and erases the capacity of the behavior to be goal-directed(Perez & Dickinson, 2020). In what follows, I review evidence that calls this idea into question and provides a new perspective on how habits can be broken and how they are learned.

## Making habits

There has been enormous progress recently in understanding habit learning from the perspective of brain science (e.g., Balleine, 2019; Balleine & O'Doherty, 2010; Robbins et al., 2019). Research indicates that the development of habit with training involves a transition from control by the dorsomedial striatum (and prelimbic prefrontal cortex) to the dorsolateral striatum (and infralimbic prefrontal cortex) (e.g., see Balleine, 2019, for one review). In contrast, there are few new ideas about the *behavioral* mechanisms that underlie habit acquisition. An early and still influential idea is Thordike’s law of effect (Thorndike, 1911), which has often been accepted as a mechanism of habit learning in discussions of the psychology of habit (e.g., Wood & Rünger, 2016). On this view, the effect of a reinforcer is to strengthen an association between the stimulus situation (S) and the response (R) that produced it. Habit is synonymous with the S-R bond. Once the bond is made, the individual will perform R reflexively when he or she encounters S. It is worth noting that this approach to habit learning is not a complete theory of instrumental learning the way psychologists historically thought it was, because it does not explain why behaviors can also be goal-directed and guided by a representation of O (as indicated by the reinforcer devaluation effect). Although S-R learning is not a general model of instrumental behavior and learning, it remains a way to conceptualize how automatic habits might be learned.

A second view of habit learning that has been entertained by learning theorists is the rate correlational view (Dickinson, 1985, 1989). On this account, actions become habits if the correlation between the rate of the behavior and the rate of reinforcement is weak. Early in learning, or when an outcome is earned on a ratio schedule of reinforcement, as the rate of the behavior increases or slows, the rate of reinforcement correspondingly increases or slows. There is thus a correlation between the rate of behavior and reward. But as the behavior rate and reward rate become more consistent and regular, particularly on interval schedules of reinforcement, there is less correlation between behavior rate and reward rate. These are conditions where action (R-O) weakens and habit (S-R) becomes strong (e.g., Perez & Dickinson, 2020). This has become a dominant idea in the learning theory of actions and habits, and it is consistent with research suggesting that habits develop more rapidly with behaviors reinforced on interval as opposed to ratio schedules (e.g., Dickinson et al., 1983). But to my knowledge, there have been surprisingly few experimental tests of whether this is what creates habit when it eventually develops with repetition and practice.

Another gap in the literature is that most of the work on animal actions and habits has been conducted with free-operant methods in which the subject is placed in a Skinner box and earns reinforcers for lever pressing on an intermittent reinforcement schedule for a long period of time (e.g., repeated sessions that are 30 min or longer). In this method, there are no immediate, local cues that set the occasion for the response. This is in contrast to habits in everyday life, which are often triggered by proximate cues (e.g., James, 1890; Wood, 2019). We know that the “context” (e.g., the Skinner box itself) can be the stimulus (S) that controls habits learned with free-operant methods (Thrailkill & Bouton, 2015)– when we test lever pressing in a different context, the habit decreases in strength. But until very recently, there was no operant research investigating habits that develop in the presence of proximate triggering stimuli.^1^ In addition to being more ecologically valid, such work would be interesting theoretically, because habit development with a discriminated operant procedure might challenge a simple rate-correlation view: When a behavior is under stimulus control, the animal responds during S (and earns reinforcers then) but not in the absence of S (earning few reinforcers then). This maintains a correlation between response rate and reinforcement rate, perhaps making habit learning difficult.

To fill the gap in knowledge, my colleagues and I (Thrailkill et al., 2018) studied habit learning using a discriminated operant method that had been used in many prior experiments (e.g., Bouton et al., 2014; Bouton et al., 2016; Colwill, 1991; Colwill & Rescorla, 1988; Rescorla, 1990, 1993, 1997). Rats pressed a lever for food pellets, but the pellets were only available on a Random Interval (RI)30-s reinforcement schedule when a 30-s stimulus (a tone) was on. Whenever the tone was on, the first response after a randomly selected interval averaging 30 s was reinforced. When the tone was off, lever pressing was not reinforced. The method produces rapid lever pressing when the stimulus is on and virtually none when it is off (rates of about 30–40responses/min in the tone and 0–10/min outside it). The question was, do rats trained this way to respond only in the presence of a trigger stimulus learn to do so out of habit? In initial experiments, we trained rats for 4, 22, or 66 sessions. Then, the rats received trials in which the reinforcing pellet Outcome was presented on its own and paired with LiCl to produce a taste aversion. (As shown in Table 1, control groups received the same pellet and LiCl, but “unpaired” on separate days.) Lever pressing was then tested, in extinction, when the tone was on and when it was off. In all cases, devaluing the reinforcer caused the rats to lever press less in the extinction tests. Thus, with as many as 66 sessions, the behavior was still a goal-directed action. Sixty-six sessions was a lot of training by most standards – it involved roughly 1,054 pairings of the response and reinforcer, whereas free-operant methods can create habit in about 360 (e.g., Thrailkill & Bouton, 2015). Despite this, the rats evidently lever pressed in S, not out of habit, but because they expected the response would lead to a pellet they valued – until it was averted by separate taste aversion conditioning.

**Fig. 1:**
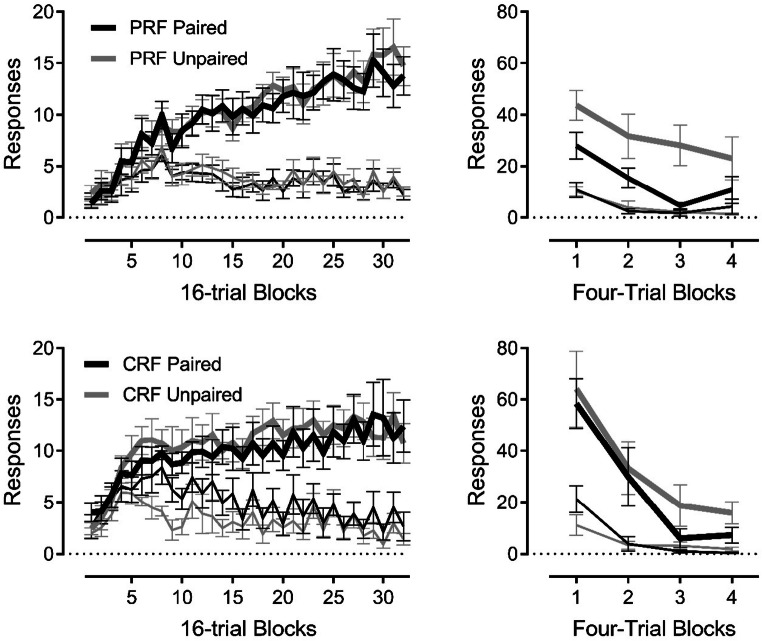
Responding during discrimination training (**left panels**) and extinction testing (**right panels**) in the Thrailkill et al. (2018) experiment described in the text. For Group CRF (continuous reinforcement, upper panels), the instrumental response was reinforced in every presentation of the discriminative stimulus (a 30-s tone), whereas in Group PRF (partial reinforcement, lower panels) it was reinforced in only half the stimulus presentations. The lowest lines in all panels indicate baseline responding outside the tone. In the extinction tests (right), responding in Group CRF was not suppressed by devaluation of the reinforcer through taste aversion conditioning (Group Paired), suggesting habit. In contrast, responding in Group PRF was suppressed by devaluation, indicating goal-directed action. Reprinted with permission of the publisher

We wondered why it was so difficult to achieve habit with our discriminated operant procedure but easy with free-operant ones. One difference was that in the free-operant case the rat carries on for a long while so that extended bouts of responding are associated with the continuous context, whereas in our discriminated method the rats only lever pressed for 30 s at a time in each tone. When we trained rats with a longer 8-min, rather than a 30-s, tone stimulus setting the occasion for lever pressing on the RI-30 schedule, habit *did* develop after extended training; rats that subsequently acquired a taste aversion to the pellet continued to lever press during the test despite their aversion to the reinforcer (Thrailkill et al., 2018, Experiments 2 and 3). But we realized that, in addition to the longer S beginning to approximate the duration of the context in the free-operant situation, the rat earned reinforcers in every 8-min S. This was not the case with the RI 30 /30-s method used before: With that combination of reinforcement schedule and S duration, the response was reinforced in only half the presentations of the tone. (Although the interval between earned reinforcers averaged 30 s, a random half of those intervals were shorter than 30 s, and half were longer. In the longer ones, the rats could lever press during a 30-s stimulus and never receive a reward.) We corrected this and compared responding in rats that could always earn at least one reinforcer in every 30-s stimulus with rats that got the usual 50% (Thrailkill et al., 2018, Experiment 4). The overall number of response-reinforcer pairings was controlled (the normal RI 30-s schedule allowed for multiple reinforcers in some trials). As illustrated at right in Fig. 1, the rats that earned a reinforcer in every S (continuous reinforcement or “CRF”) learned a habit (their responding was unaffected by reinforcer devaluation), and those that earned a reinforcer in only 50% of the Ss (partial reinforcement or “PRF”) showed action (the response was suppressed by the devaluation). In a discriminated operant procedure, habit developed when the reinforcer was predictably delivered in every S, but not when it was unpredictably delivered in only half of them. Notice that, in everyday human habits (e.g., smoking), responses to a trigger stimulus are probably also predictably reinforced in every stimulus.^2^

The reason we tested the role of reinforcer predictability is that we knew it can have powerful effects on attention processes. For example, according to at least one venerable theory of associative learning, the Pearce-Hall model of Pavlovian learning (Pearce & Hall, 1980), attention paid to cues that predict a reinforcer should decline as the reinforcer becomes better and better predicted during extended conditioning. Why should we pay attention to something once we know what it means? (We should respond to it automatically.) On the other hand, if the cue is paired with a reinforcer on only a random half of its presentations, the reinforcer (or lack of it) is to some extent surprising on every trial. According to the theory, 50% reinforcement will maintain attention to the imperfect predictor (as well as all other cues or events that are present on the trials). The results of experiments in rats (e.g., Kaye & Pearce, 1984; Wilson et al., 1992) and humans (Hogarth et al., 2008) suggest that a visual cue that has an uncertain (50%) relation to an outcome maintains orienting behavior, whereas a cue that has a perfect (100%) relation to an outcome does not. Applied to habit learning, our idea was that attention to what we are doing might likewise decline as the reinforcer becomes predictable. And a behavior that we are doing without attending to it is one we are doing out of habit. Thus, when the reinforcer is imperfectly predicted by S, we might pay more attention to both the predictor and the behavior itself. That may be the reason why a discriminated operant that is reinforced during only some of the S presentations may keep the status of goal-directed action.

To further test this idea, we (Thrailkill et al., 2021) went on to adapt a second method that Vandaele et al. (2017) had independently shown also creates a discriminated habit. Vandaele et al. also studied rats lever pressing, but in their method, the lever was normally retracted from the Skinner box. Whenever the lever was inserted into the chamber, the rat was reinforced the fifth time it pressed it. (The lever was then retracted again at the same time the reinforcing pellet was delivered.) Lever insertion was thus the cue to begin responding. This method produced a habit, as suggested by the results of reinforcer devaluation tests. Was it possible that habit development here was also due to decreased attention to the behavior as the reinforcer was predicted by the lever-insertion stimulus? Vandaele et al. had suggested that lever-insertion provides an unusually salient cue; perhaps that was what allowed the response to become habitual. To test the idea, we replicated their lever-insertion procedure and compared it to one in which the lever was always in the box, but the fifth response on it was reinforced whenever a tone came on. (Like the lever in the lever-insertion group, the tone was also withdrawn when a reinforcer was earned). With a moderate amount of instrumental training, lever pressing occasioned by either lever or tone was goal-directed, as indicated by our reinforcer devaluation tests. But with more extended training, responding occasioned by either kind of cue also became a habit. That is, the rats continued to press the lever in extinction after a taste aversion had been conditioned to the pellet on its own.

**Fig. 2:**
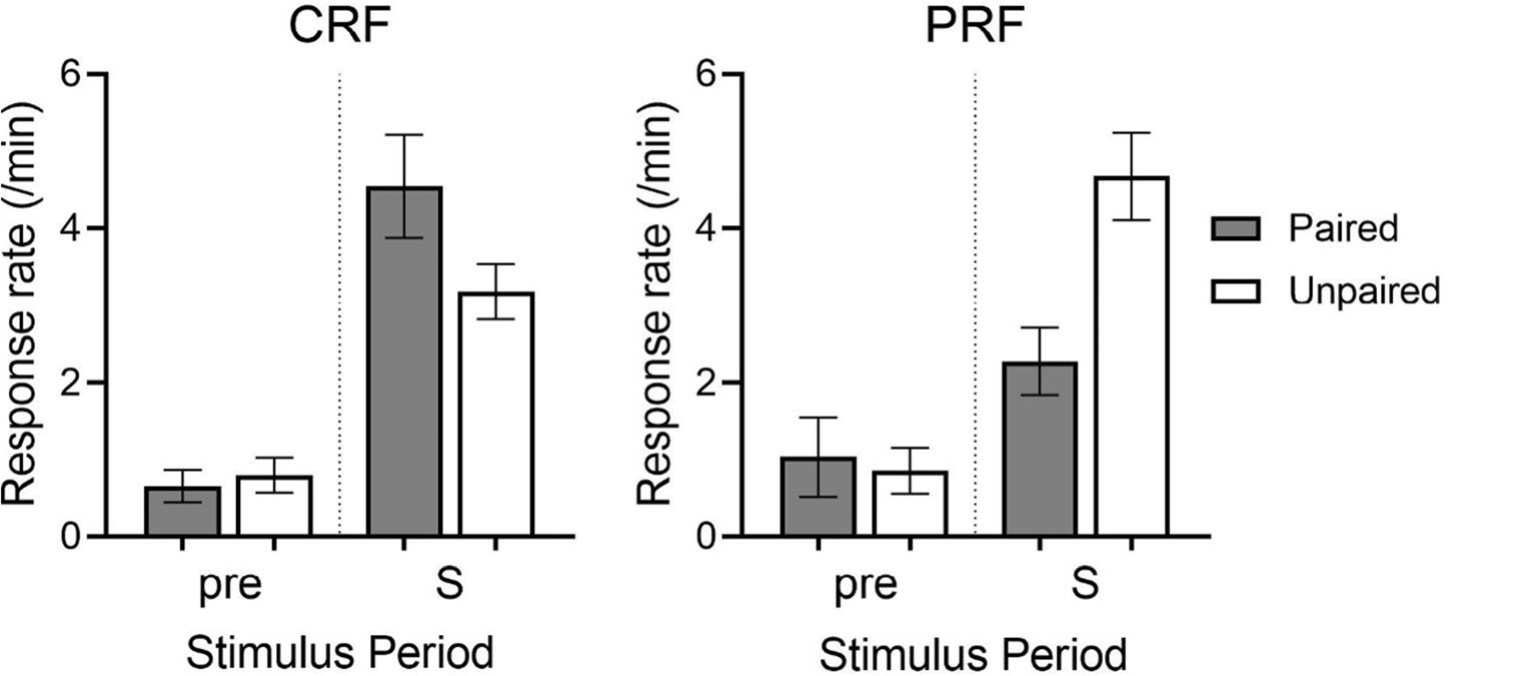
Responding during extinction testing in the Thrailkill et al. (2021) experiments described in the text. For Group CRF (continuous reinforcement), the instrumental response had been reinforced in every presentation of the discriminative stimulus (a brief 6-s tone), whereas in Group PRF (partial reinforcement) it had been reinforced in only half of the stimulus presentations. Responding in Group CRF was not suppressed by separate taste aversion conditioning of the reinforcer (Group Paired), indicating habit; in contrast, responding in Group PRF was suppressed, indicating goal-directed action. “pre” indicates baseline responding outside the tone. Adapted from Thrailkill et al. (2021) and used with permission of the publisher

We then asked whether we could prevent this habit learning by reducing the reinforcer’s predictability – as we had in the previous work (Thrailkill et al., 2018). Two groups were presented with a series of trials in which the tone was presented for 6 s. (The short stimulus duration roughly matched the duration of the trials that developed in the first experiment.) In one group, every time the tone came on, the response was reinforced on a VI 3-s schedule (again based on what developed in the first experiment). In another group, the response was reinforced in only half of the tone presentations. The CRF and PRF groups were equated on the number of trials on which responding was reinforced. In one experiment the PRF group received non-reinforced trials between the reinforced trials scheduled at the same intervals as the CRF group; in another experiment, we presented tones with the same intervals between them, but we eliminated reinforcement on half the trials. Figure 2 shows the results averaged over the two experiments. As Thrailkill et al. (2018) had found with stimuli that were five times as long and a reinforcement schedule that was 1/10 as rich, the CRF procedure produced habit, whereas PRF maintained goal-direction. Habit develops when the cue always predicts the response will be reinforced, but not when the cue is associated with a reinforcer unpredictably on a random half of the trials.

We went on to study the PRF/CRF difference in a final experiment that used lever-insertion as the stimulus instead of the tone. The earlier experiment had suggested that rats learned to respond to lever-insertion more quickly than to the tone, perhaps because it was indeed the more salient stimulus (e.g., Holland et al., 2014; Vandaele et al., 2017). Although the two stimuli had been similar in controlling action and habit after moderate versus extended instrumental training *when the response had been reinforced in every stimulus presentation*, could the more salient lever be less affected by reinforcer predictability? It turns out that it was. When we compared CRF and PRF procedures with lever insertion, habit developed with either of them. We do not have a complete understanding of the difference between lever insertion and the tone.^3^ But with lever insertion, there was little difference in lever-pressing rate with the PRF and CRF procedures, as if non-reinforcement had less impact on responding in the presence of this stimulus.

The results of these experiments suggest that habits do develop in animals with discriminated operant methods that give a role to proximate cues. With a standard tone stimulus, habit develops if the tone predicts that a reinforcer will occur whenever it is presented, but not when reinforcer delivery is uncertain (Thrailkill et al., 2018, 2021). With a lever-insertion stimulus, which may be more salient, habit does develop even when the reinforcer is uncertain, perhaps because the stimulus (and the strength of its presumed prediction of the reinforcer) is less decremented by the non-reinforced trials (Thrailkill et al., 2021). If one is interested in developing a healthy habit, such as wearing sunscreen or regular exercise, try reinforcing it consistently and predictably in the presence of a consistent stimulus. As an additional hedge, make that stimulus robust and salient.

## Breaking habits

The results regarding the creation of habits may have implications for how they might be broken after they are learned. Recall that many intuitions about addiction assume that habits are rigid and hard to change, a rather fixed result of repetition and practice. On the other hand, the attentional perspective, which holds that a behavior becomes a habit once you aren’t attending to it, suggests more flexibility: A behavior’s status as habit or action might in principle change as attention changes. The idea seems consistent with my own experience of my everyday habits, which seem to pop in and out of habit and action mode. For instance, I was driving to the lab one day (out of habit?) only to realize that it was Sunday and my goal was actually getting to the grocery store. I was able to recover – and get to the grocery store – once I realized my mistake. Thus, in normal life it seems possible to switch a behavior initiated out of habit back to action status. It turns out that something like this might be true for the rat, too.

### Unexpected reinforcers

**Fig. 3:**
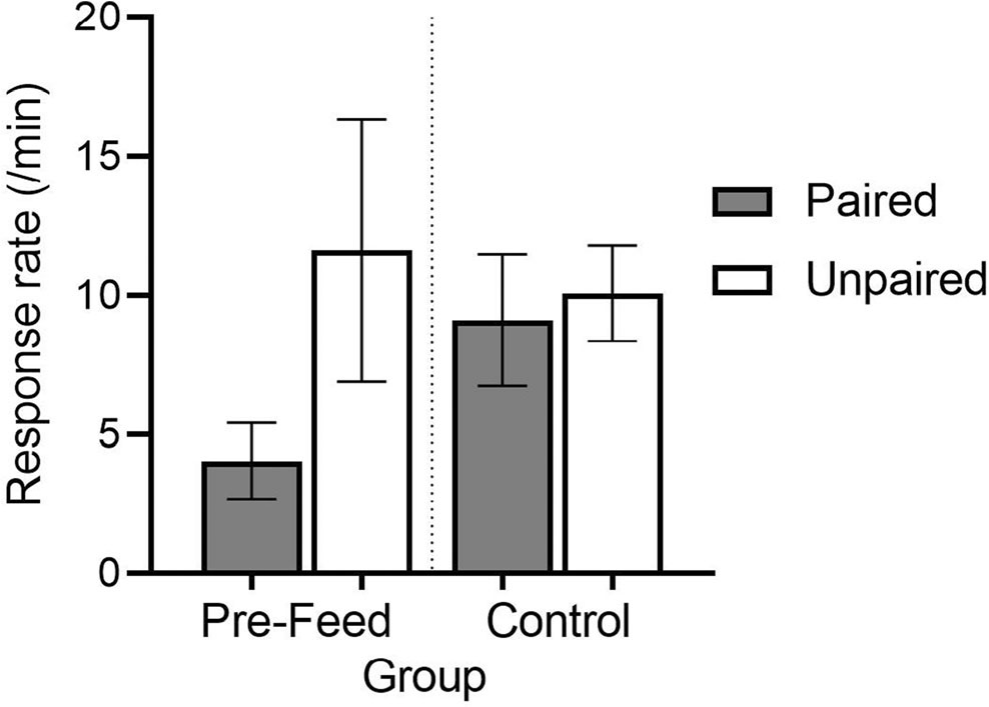
Instrumental responding during extinction testing after the reinforcer was devalued (Paired groups) in an experiment by Bouton et al. (2020) described in the text. In the Control group, reinforcer devaluation had no effect on responding, indicating that the response was a habit. However, in the group given pre-feeding with an irrelevant food pellet just before the test (Pre-Feed), the response was an action again. Adapted from Bouton et al. (2020) and used with permission of the publisher

In previous tests of the Pearce-Hall model’s attention rule, researchers had shown that depressed attention to a conditioned stimulus can be re-engaged by associating it with a surprising outcome (e.g., Kaye & Pearce, 1984; Pearce & Hall, 1982; Wilson et al., 1992). If habit learning follows a similar rule, then we should likewise be able to re-engageattention and return a behavior from habit to action by introducing a surprising outcome. Bouton et al. (2020) tested this idea and found that it worked. Rats received extended free-operant lever-press training (RI 30) with either a grain-pellet or a sucrose-pellet reinforcer. Then, half the rats received a single session in which the usual grain pellet was switched to sucrose or sucrose was switched to grain. The other half (control subjects) received a typical session in which lever pressing continued to earn the usual reinforcer. The first reinforcer was then devalued, as usual, by pairing it (or unpairing it) with LiCl in the standard design (Table 1). In the controls, lever pressing was not affected by the devaluation treatment, confirming that the response had become a habit. But after an unexpected change in reinforcer type, the lever-press response was affected by devaluation, suggesting that it had returned to action status. Further results suggested that the surprising reinforcer does not have to be contingent on the response. Bouton et al. (2020, Experiment 3) gave rats enough lever-press training to create a habit and then devalued the reinforcer with taste-aversion learning (the reinforcing pellet was paired or unpaired with LiCl). The devaluation effect had no effect on lever pressing in controls (Fig. 3), indicating that lever pressing was a habit. But experimental subjects received an unexpected 30-min pre-feeding with an irrelevant food pellet in the home cage just before the test. As can be seen, this treatment converted the habit evident in the controls back to a goal-directed action that was depressed by the devalued reinforcer.^4^ In both of these experiments, the behavior’s status as a habit was not permanent; it could readily switch back to a goal-directed action again.

A separate line of research also suggested that habits can readily return to action status (Trask et al., 2020). These experiments followed a previous report that had studied the effects of inactivating either the prelimbic or infralimbic cortex on overtrained and moderately trained instrumental behavior (Shipman et al., 2018). In that research, one response (R1) was extensively trained in one context (Context A), and then, at the end of training, a new response (R2) was introduced and trained in several interleaved sessions conducted in Context B. Either response (lever pressing or chain pulling, counterbalanced) was reinforced on our usual RI 30-s schedule. The reinforcer was then devalued via taste aversion in both contexts (controls received the pellets and LiCl unpaired, as in Table 1) before R1 and R2 were tested in extinction. In one experiment, half the rats had the prelimbic cortex inactivated pharmacologically (by delivering baclofen/muscimol directly there); in the other experiment, the infralimbic cortex was inactivated. We found that inactivation of the prelimbic cortex suppressed moderately trained (but not extensively trained) instrumental behavior, and that inactivation of infralimbic cortex suppressed the extensively trained (but not moderately trained) response. The double dissociation was consistent with previous work suggesting that the prelimbic cortex controls moderately trained actions and infralimbic controls extensively trained habits (e.g., Coutureau & Killcross, 2003). But there was a rub: The effects of reinforcer devaluation suggested that both the moderately trained R2 and extensively trained R1 were goal-directed actions. (They were both suppressed by reinforcer devaluation.) Thus, a more accurate description of the infralimbic cortex’s role might be that it is important when a behavior (either habit or action) has been extensively trained.

Given our other evidence suggesting that habits can convert to action (Bouton et al., 2020), we entertained the idea that the overtrained R1 in the Shipman et al. (2018) experiments had achieved habit status during training, but something within the training protocol had switched it back to action. The unexpected interleaved training of R2 in Context B at the end of R1 training seemed the likely candidate. And in a series of experiments, Trask et al. (2020) established that this was in fact the variable that caused the conversion. When R1 was trained the same way but without the interleaved R2 training, or when R2 training began after R1 training had finished, reinforcer devaluation tests determined that R1 was indeed a habit. Additional experiments found that the interleaved training of R2 could be reinforced by a different reinforcer (sucrose when grain had been trained or grain when sucrose had been trained). And it turned out that R2 was not necessary; all we needed to do was present the reinforcers freely (not contingent on any response) during the sessions in Context B. Thus, the causal factor seemed to be the unexpected reinforcers delivered in Context B during sessions interleaved with the final R1 sessions in Context A. These were enough to convert a habitual R1 back to action. The findings were clearly similar to those of Bouton et al. (2020).

The findings just described made it clear that, with our methods, the status of a behavior as a habit is not fixed or permanent. A habit can readily return to the goal-directed state. One implication is that habit learning does not erase the original action learning. And the findings provided a potential reason why habits have been difficult to observe in some human laboratory experiments. Although Tricomi et al. (2009) found that overtraining a simple operant response in humans can create a habit (as judged by insensitivity to reinforcer devaluation), de Wit et al. (2018) later reported failures to demonstrate habit in several experiments. Although they concluded that they had failed to “induce” a habit, our results suggest another possibility: Perhaps habit had been successfully created, but some unidentified feature of the procedure had switched it back to action.

### Effects of context change

The impermanence of habit and the recovery of action is also consistent with other work in which we have manipulated context change (Steinfeld & Bouton, 2020, 2021). We became interested in context change when we began asking whether actions and habits are similarly influenced by extinction, the process in which a behavior is weakened when it is no longer reinforced. Extinction is a process that my laboratory has studied for many years. We and others have shown that extinction weakens instrumental responding, but does not erase the original learning. Instead, extinction involves a form of context-specific inhibition (rather than unlearning) of the response (e.g., Bouton, 1993, 2019; Bouton, Maren, & McNally, 2021b). For example, we have performed many studies of the *renewal effect*: If the context is changed after extinction has been learned, the original response can return. Steinfeld and I were interested in whether actions and habits are affected the same way by extinction. Are actions and habits both renewed after extinction when the context changes? And do actions renew as actions, and habits renew as habits?

To answer these questions, Steinfeld and Bouton (2020) ran a number of experiments that involved context switches after action, habit, and extinction learning. Our contexts, as in all our prior work, were different sets of Skinner boxes that were housed in different locations in the lab and had different olfactory, visual, and tactile features. In one pair of experiments, we gave lever pressing either a small amount of training on our usual RI 30-s reinforcement schedule (to create a goal-directed action) or an extensive amount of training (to create a habit) in one context, Context A. Then the reinforcer was devalued by pairing it with LiCl in that context as well as a second context (Context B).^5^ (As usual, experimental subjects had the pellet and LiCl paired and controls had pellet and LiCl unpaired.) In the next phase, lever pressing was extinguished in the second context (Context B) by allowing the rat to perform it without reinforcement. Once responding had reached approximately zero, we tested it back in the original training context (Context A). In either case, responding returned (was “renewed”), and by comparing the groups for which the pellets had been devalued or not, we could see that the response that had been trained as an action renewed as action and the response trained as a habit renewed as a habit. Thus, actions and habits both return to their pre-extinction status in their original training context after extinction in another one (“ABA renewal”).

In subsequent experiments, we tested for renewal in a different context using the so-called “ABC renewal” design. Here rats received either (minimal) action training or (extensive) habit training (with RI 30 again) in Context A, had the reinforcer devalued, and then had the response extinguished in Context B. In contrast to the previous experiments, here we tested for renewal in a neutral third context, Context C. (I should mention that we had devalued the reinforcer in all three contexts before extinction began.) Notice that Context C is different from both the context where lever pressing had been acquired (Context A) and where it had been extinguished (Context B). Remarkably, regardless of whether the behavior had been trained as an action or habit in Context A, it renewed as an *action* in Context C. Extinction was context-specific, as it always is. But so was habit; when the habit trained in A was tested in C, it was goal-directed again. The result was actually consistent with what we had seen during all the extinction phases, where lever pressing was always extinguished in a different context (B) after training in A. There we also always found evidence of conversion to action – the pellet-LiCl pairings suppressed behavior there relative to unpaired pellet/LiCl. In contrast, an action trained in A expressed as an action in B or C. Habits are context-specific, but actions are not (see also Thrailkill & Bouton, 2015).

**Fig. 4:**
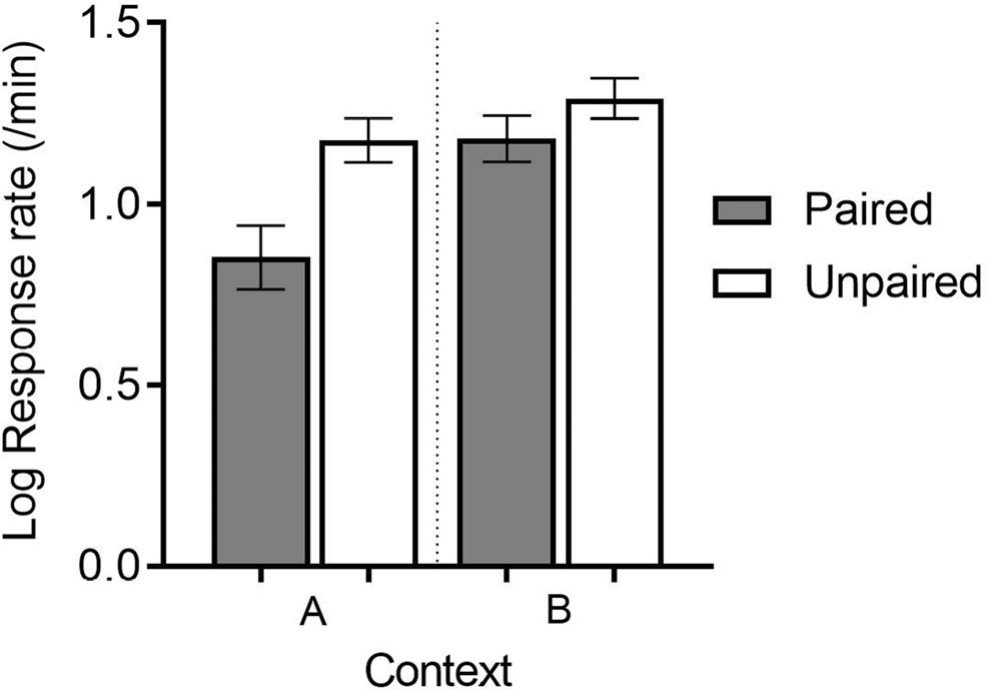
Results of extinction testing after reinforcer devaluation (Paired group) in an experiment by Steinfeld and Bouton (2021) described in the text. Rats had received a small amount of instrumental training in Context A before the response received more extended training in Context B prior to reinforcer devaluation. Results suggest that the response was a habit in Context B, but that goal-direction was renewed in Context A. Adapted from Steinfeld and Bouton (2021) and used with permission of the publisher

This possibility received more support from additional experiments (Steinfeld & Bouton, 2021). Instead of studying extinction, these experiments merely investigated the contextual control of action and habit after the action was converted to habit by extended training. Does habit learning merely interfere with the expression of goal-directed action, just as extinction learning interferes with conditioning? In one experiment, we gave rats a modest amount of lever-press training with a RI 30-s reinforcement schedule in Context A (to establish it as an action there) and then converted it into a habit by giving it more extended training in Context B. When the reinforcer was devalued in both contexts, tests demonstrated that lever pressing was still a habit in Context B, but switched to action when tested in Context A (the original action context) (see Fig. 4). In another experiment, after action training in A and conversion to habit in B, we tested the response in B and in a third context, Context C. (Reinforcer devaluation was conducted in all three contexts before the test.) Here the response retained its habit properties in B, but expressed as an action in Context C. The simplest interpretation of these results after action➔habit conversion is that goal-direction transfers well across contexts (i.e., from A to C), whereas habit does not transfer as effectively from B to C. In our animals and with our methods, habit learning does not seem permanent, but like extinction, interferes with goal-direction in a context-specific way.

The emerging parallel between habit and extinction learning could use further discussion. Over the years, our extinction research has documented that extinction does not erase what has been learned before it, but instead interferes with it. A number of phenomena are consistent with this perspective (see Bouton, 2017, 2019; Bouton, Maren, & McNally, 2021b). If the reinforcer is presented freely after extinction, it can “reinstate” an extinguished response (e.g., Baker, 1990; Rescorla & Skucy, 1969; Winterbauer & Winterbauer & Bouton, 2011); if time is allowed to pass after extinction, the extinguished behavior can “spontaneously recover” (e.g., Rescorla, 2004); and if we change the context after extinction, the behavior renews – we have observed ABA, ABC, and even AAB forms of renewal, where the first, second, and third letters denote the contexts of conditioning, extinction, and testing (e.g., Bouton et al., 2011). The ABC and AAB forms of renewal are especially interesting, because they suggest that the second-thing learned (extinction) is more context-specific than the first-thing learned (acquisition). This turns out to be true of many “retroactive interference” treatments in both instrumental and Pavlovian conditioning (e.g., Bouton et al., 2021b). For instance, in instrumental learning, a behavior that has been punished by presenting a response-contingent footshock is also reinstated with free presentations of the original reinforcer (e.g., Panlilio et al., 2003), recovers with the passage of time (Estes, 1944), and is renewed by a change of context (e.g., Bouton & Schepers, 2015). The parallel with habit learning is apparent: In the action➔habit conversion, like the conditioning➔extinction (or conditioning➔punishment) conversion, the first-thing learned (goal-directed action) remains at least partially intact after second learning. It also transfers relatively well across contexts. In contrast, the second-thing learned (habit) does not. Although a little S-R learning may occur early during instrumental training (e.g., Thrailkill & Bouton, 2015), the action➔habit transition seems to fit a general organizing principle of associative learning.

The picture that emerges in our studies of action➔habit conversion is one of flexibility rather than rigidity. Habits are not really fixed or confining. As noted earlier, the development of a habit and automaticity is theoretically functional because it preserves limited space in working memory for processing other things. The fact that action remains available for expression after habit learning allows even more flexibility and adaptability. Although actions seem less affected by context change than habits are, the action➔habit conversion and context-dependent interference by habit allows the organism to switch between the two with changes in context (see below). And when a habit enters a new situation (i.e., the context changes), it makes functional sense for the behavior to be goal-directed again so that the animal can learn what goals are available there (new R-Os). When a behavior is in habit mode, it exploits the tried and true; when it is in action mode, it can presumably explore new opportunities or connections (cf. Beeseley et al., 2015)

## Discussion

The findings just summarized are so consistent with other types of retroactive interference effects in animal learning that it seems worth remembering that they challenge common intuitions about habit learning that view it is a fixed behavioral endpoint. It should be noted that the dual-process or “associative-cybernetic” approaches of Dickinson and Balleine and colleagues (e.g., Balleine & Ostlund, 2007; de Wit & Dickinson, 2009; Dickinson, 2012) do posit separate and co-existing action and habit memories. Although there has been relatively emphasis on the recovery of action performance after habit has been learned (but see Balleine & Dezfouli, 2019; Balleine et al., 2009), the views are consistent with results suggesting that certain neural manipulations, in addition to the behavioral manipulations I reviewed here, can restore goal direction when the circuits controlling habit are suppressed (e.g., Coutureau & Killcross, 2003; Yin, Knowlton, & Balleine, 2006). Other frameworks that emphasize multiple parallel memory systems in the brain (e.g., Gruber & McDonald, 2012; McDonald & White, 1993; White, 1996) also allow behavioral flexibility. Yet in one of the most recent statements of the dual-process theory (and the rate correlation theory of action/habit acquisition), Perez and Dickinson (2020) explicitly assume that habit erases action knowledge when it is learned. It is hard to see how that perspective can allow actions to recover with surprising reinforcers or with context change without some significant modification.

Approaches to action and habit in the reinforcement learning tradition (e.g., Sutton & Barto, 1998) have also often accepted the coexistence of action and habit systems and allowed one or the other to be selected by a hypothetical “arbitrator” for behavioral control (e.g.,Daw et al., 2005 ; Lee et al., 2014). Actions or behaviors based on a “model-based” system (which encodes representations of all the behavior’s possible consequences) are considered rich but computationally expensive, while habits or behaviors based on a “model-free” system (which encodes only the behavior’s “cached” or average value) may be efficient but inflexible. Given the tradeoff, the arbitrator selects between the two systems according to various criteria, such as the accuracy or prediction error connected with each (Daw et al., 2005; Lee et al., 2014) or the prevailing rate of reinforcement (Dezfouli et al., 2014). It remains to be seen whether reinforcement learning models can address the present results, which imply a fundamental role of the *context* in selecting between action and habit. And it is also not clear whether existing reinforcement learning theories can accommodate the evidence that, with an equal number of response-reinforcer pairings, habit develops when the reinforcers are predictable, and not when they are not (Thrailkill et al., 2018, 2021).

**Fig. 5:**
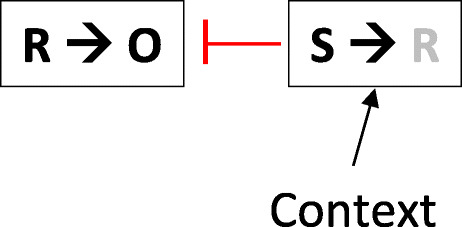
A hypothetical action-habit switch. See text for explanation

Figure 5 provides a simple description of how habit and goal-direction might interrelate that is loosely inspired by our understanding of extinction (e.g., Bouton, 1993, 2019; Bouton, Maren, & McNally, 2021b) and retroactive interference processes more generally (e.g., Miller, 2021). It can be seen as a dual-process approach that emphasizes retrieval and inhibition processes. When an instrumental behavior is learned and then repeatedly practiced, the organism learns and retains two things. Initially, the response is mostly associated with O (R-O), and is therefore goal-directed and sensitive to O’s value. As practice continues, and the reinforcing O becomes predictable, an S-R association begins to dominate. However, instead of erasing R-O, S-R exists along with it, coded by a separate circuit in the brain (e.g., Balleine, 2019; Balleine & O'Doherty, 2010; Gruber & McDonald, 2012; Robbins et al., 2019). Crucially, the activation of S-R is context-dependent, either because the context itself provides the S associated with R, or because (as we have seen in other retroactive interference paradigms) the context controls the second thing learned (e.g., Bouton, 1993; Nelson, 2002). Equally important, activation of S-R suppresses or inhibits R-O. The figure shows the R in S-R with a lighter color to capture the idea that an R evoked by S requires little attention to perform. The result of this simple mechanism is that in the habit context, the habit association is active and action is suppressed, whereas outside the habit context, S-R is not activated, and goal-direction(R-O) is expressed. It is worth noting that the range of cues that can constitute context is arguably very wide, potentially including time, drug states, deprivation and stress states, and other behaviors among other things (e.g., Bouton, 2019; Bouton et al., 2021a, b). To explain why the presentation of surprising reinforcers also seems to activate action after habit learning (Bouton et al., 2020; Trask et al., 2020), we could thus note that they may provide a form of context change (e.g., Bouton et al., 2021b). Alternatively, but perhaps more speculatively, surprising food reinforcers may restore attention generally to appetitive behaviors that have led to them, and recovered attention to an R may be sufficient for R to activate O and become goal-directed again.

The flexibility of the switch between habit and action seems inconsistent with a simple view of addiction as the acquisition of a rigid habit. Habit is not a fixed endpoint; its acquisition does not erase R-O and make it permanently unavailable. If habits do play a role in addiction, they may do so because the habit-action switch becomes sticky (see also Balleine et al., 2009; Lee et al., 2014). As a consequence, once a habit is learned, it may be difficult to switch it back to goal-directed status. On the other hand, goal-direction processes themselves may play a major role in addiction (e.g., Hogarth, 2020). We need more research and information on the processes and mechanisms that might underlie the habit-action switch (see Corbit et al., 2014; Furlong et al., 2015).

The other idea that the reinforcer must be predictable in order to create habit (Thrailkill et al., 2018, 2021) seems to lie outside the scope of existing habit theories. As noted earlier, the importance of predictability is not anticipated by the law of effect, the response/reinforcer rate correlation view, or existing models of reinforcement learning. But just as prior research on retroactive interference in associative learning (e.g., extinction) might provide some new insight, previous research on attention in associative learning might provide additional perspective here. The attention explanation given above is based on a well-known theory of attention and its role in Pavlovian learning (Pearce & Hall, 1980). To repeat, as an action’s reinforcer becomes better and better predicted, attention to the response (as well as to the predictive discriminative stimuli) is expected to decline. Extended practice will thus normally decrease attention to the response; when this occurs, habit is created, and the response can be elicited by S without the organism processing O. In our experiments (Thrailkill et al., 2018, 2021), attention to the response was theoretically maintained by making the reinforcer uncertain from trial to trial; this maintained goal-direction and prevented habit. The correspondence between our findings in instrumental learning and attentional decline and restoration in classical conditioning (and human learning) seems clear. Outcome uncertainty maintains attention to behavior and therefore action, whereas outcome predictability reduces it and creates habit.^6^

Attention to behavior could also work in a different way. Although the arrangement in which S predicted the reinforcer on 50% of its presentations (Thrailkill et al., 2018, 2021) reminded us of prior tests of the Pearce-Hall model (e.g., Kaye & Pearce, 1984), there is another way to think about the results. In a different account of attention and learning, Mackintosh (1975) argued that attention increases (rather than decreases) to the best predictor of a reinforcer. Thus, if there is a good predictor, the organism will tune it in rather than tune it out. Crucially, the organism will also tune out contemporaneous predictors that are not as good as (or no better than) the best one. Because of this, the Mackintosh attention rule provides another way to think about the Thrailkill et al. (2018, 2021) findings: When S always predicts a reinforcer, the organism should tune it in and tune out other predictors—including, perhaps, the response. In contrast, when S is a weaker predictor, as when it predicts a reinforcer only 50% of the time, the organism may be less inclined to tune out the response – thus maintaining goal direction rather than habit. On this view, instrumental responding could become automatic and habitual whenever the triggering S is a strong and reliable predictor of the reinforcer.

Mackintosh’s attention rule is consistent with a number of results (e.g., Le Pelley et al., 2016; Pearce & Mackintosh, 2010), and contemporary approaches to attention and learning now suppose that both the Mackintosh and Pearce-Hall rules can play a role in any situation (e.g., Le Pelley, 2004; Pearce & Mackintosh, 2010; see Chao et al., 2021, for recent discussion). The Mackintosh rule is worth mentioning because it can also explain another finding, noted earlier, that some researchers see as central to the action/habit distinction: Habits appear to develop more readily when responding is reinforced on interval than on ratio reinforcement schedules (e.g., Dickinson et al., 1983). In ratio schedules, the organism must make a number of responses to earn each reinforcer, whereas in interval schedules, the response is reinforced when it is made after some interval of time has elapsed. With a ratio schedule, the higher the rate of responding, the higher the rate of reinforcement, and response rate is thus a good predictor of the reinforcer. The Pearce-Hall rule predicts that attention to the response will decline – and thus incorrectly implies habit. But according to Mackintosh, attention may be drawn to the predictive ratio-reinforced response, maintaining goal direction. In contrast, on an interval schedule, the rate of responding is not as good a predictor of reinforcement rate, and other background cues (such as the context) may be as good at predicting it – thus allowing attention to the behavior to decline. The difference between ratio and interval schedules in generating habit may well be consistent with an attentional view.

Still other theories of associative learning are worth mentioning. Many emphasize that processing the *reinforcer* declines during conditioning, in contrast to the processing of events (cues or behaviors) that predict it. For example, the Rescorla-Wagner model (Rescorla & Wagner, 1972) and its extensions by Wagner (e.g., Wagner, 1978, 1981, 2008; Wagner & Brandon, 1989) emphasize that the reinforcer becomes less surprising and less processed as classical conditioning approaches asymptote. If an instrumental reinforcer were likewise less processed after asymptotic training, it could provide a different reason why reinforcer devaluation becomes ineffective: Reinforcer devaluation will not affect instrumental responding if the animal is not processing the reinforcer. The main problem with this approach is that it predicts that the effect of devaluing the reinforcer will wane as either instrumental or classical conditioning approaches asymptote. Although things work this way in instrumental learning (i.e., habit is eventually observed), classically conditioned responding is still suppressed by reinforcer devaluation even after extensive training (e.g., Holland, 1990, 2005). As far as we currently know, there is no point at which the Pavlovian response becomes unaffected by reinforcer devaluation. This makes me reluctant to think that reduced processing of the reinforcer, as opposed to reduced processing of the response, can provide a satisfactory account of habit development.

Perhaps the habit context mostly modulates attention to the response instead of switching between S-R and R-O. On this account, the switch back to R-O after unexpected reinforcers or context change (Bouton et al., 2020; Steinfeld & Bouton, 2021; Trask et al., 2020) would be created by merely increasing attention to R (and the consequent associative retrieval of O). We are a long way from a complete understanding of how habits are learned and how they operate. But the research reviewed here emphasizes that goal-direction is not erased during habit learning, and that it can be recovered under the right conditions. It also suggests that habits develop when the reinforcer becomes well-predicted by S, as if the organism pays less attention to the response. The two conclusions are consistent with what we know about interference and attention in associative learning, which until now have had too little contact with the science of goal-directed actions and habits.

## Footnotes

1 There is a rich literature demonstrating S-R learning in mazes (as opposed to operant chambers) using discrete-trial tasks (e.g., McDonald & White, 1993; Packard & McGaugh, 1996). Although these tasks and others can elegantly separate S-R learning from other types of learning (e.g., place learning), it is not necessarily clear that an S-R response is habitual in the sense that I am using the term unless the response is also shown to persist despite reinforcer devaluation (e.g., Sage & Knowlton, 2000; Smith et al., 2012). For example, instead of being habitual, the S-R association might be embedded in a hierarchical S-(R-O) structure in which a *goal-directed* R is occasioned (rather than elicited) by the S (e.g., Colwill & Rescorla, 1990; Trask & Bouton, 2014).

2 It is also worth noting that the PRF group was slower than the CRF group to extinguish its responding during the extinction test. This is the familiar “partial reinforcement extinction effect.” What makes it interesting here is that a partially reinforced action was evidently slower to extinguish than a continuously reinforced habit, putting the ostensible rigidity and inflexibility of habits in new perspective.

3 We also considered the possibility that the lever-insertion procedure had encouraged lever pressing in the form of a Pavlovian “sign-tracking” response (e.g., Saunders & Robinson, 2013). One thing weighing against this possibility is that after extensive training, Pavlovian sign-tracking (but not our instrumental lever pressing) remains sensitive to reinforcer devaluation through taste-aversion conditioning when the taste aversion conditioning is conducted in the same context (e.g., Amaya et al., 2020).

4 Although the food pellets the rats were pre-fed with prior to the test were relatively novel, a possible implication of this finding is that the specific satiety method of devaluing a reinforcer (which involves pre-feeding the reinforcing food pellet to satiety before the test, e.g., Colwill & Rescorla, 1985; Dickinson et al., 1995) can be problematic as a measure of habit. This does seem true in my own laboratory, where we have had no success in using the method to demonstrate habits (e.g., Bouton et al., 2020, Experiment 2), as if the pre-feeding always turned habit back to action again. Specific satiety remains a good measure of goal direction, though.

5 We devalued the reinforcer in both contexts because there is evidence that the effects of reinforcer devaluation through taste-aversion learning are specific to the context in which aversion conditioning occurs (Bouton et al., 2021a). Testing was to occur in both contexts.

6 The results of some experiments suggest that habit is more likely to develop when responding is reinforced on a variable interval (VI) reinforcement schedule compared to a fixed-interval(FI) schedule (DeRusso et al., 2010; Garr et al., 2020). Because the reinforcer is more predictable when its timing is fixed compared to variable, the result seems to contradict the view presented here. However, as noted by Thrailkill et al. (2021), the problem with the VI-FI comparison is that animals cluster their responding near the reinforcer on fixed-interval schedules (i.e., the behavior “scallops”) but distribute responding more evenly over time on variable ones. This confounds “predictability” with the average interval between the response and outcome – and shorter intervals (as in fixed interval) yield goal-direction instead of habit learning (Urcelay & Jonkman, 2019). Our experiments (Thrailkill et al., 2018; Thrailkill et al., 2021) manipulated predictability based on what the S predicted on each trial. There was no similar confound between the average R-O interval and the predictable and uncertain reinforcers.
